# How Parents Read Counting Books and Non-numerical Books to Their Preverbal Infants: An Observational Study

**DOI:** 10.3389/fpsyg.2016.01100

**Published:** 2016-07-21

**Authors:** Alison Goldstein, Thomas Cole, Sara Cordes

**Affiliations:** ^1^Department of Psychological and Brain Sciences, University of DelawareNewark, DE, USA; ^2^Department of Psychology, Boston CollegeChestnut Hill, MA, USA

**Keywords:** numerical development, counting, numerical input, counting books, cardinality

## Abstract

Studies have stressed the importance of counting with children to promote formal numeracy abilities; however, little work has investigated when parents begin to engage in this behavior with their young children. In the current study, we investigated whether parents elaborated on numerical information when reading a counting book to their preverbal infants and whether developmental differences in numerical input exist even in the 1st year of life. Parents and their 5–10 months old infants were asked to read, as they would at home, two books to their infants: a counting book and another book that did not have numerical content. Parents’ spontaneous statements rarely focused on number and those that did consisted primarily of counting, with little emphasis on labeling the cardinality of the set. However, developmental differences were observed even in this age range, such that parents were more likely to make numerical utterances when reading to older infants. Together, results are the first to characterize naturalistic reading behaviors between parents and their preverbal infants in the context of counting books, suggesting that although counting books promote numerical language in parents, infants still receive very little in the way of numerical input before the end of the 1st year of life. While little is known regarding the impact of number talk on the cognitive development of young infants, the current results may guide future work in this area by providing the first assessment of the characteristics of parental numerical input to preverbal infants.

## Introduction

While in the past two decades, a rich literature has emerged regarding how human infants process non-symbolic numerical information (e.g., detecting a difference between 8 and 16 dots; Xu and Spelke, 2000; Libertus and Brannon, 2010; see Anderson and Cordes, 2013), very little work has centered on whether (and if so, how early) preverbal infants acquire an understanding of our formal symbolic number system, such as learning the rules of verbal counting (but see Slaughter et al., 2011). Importantly, early counting abilities are critical for later success in formal mathematics (Duncan et al., 2007; Passolunghi et al., 2007; Stock et al., 2009; Geary, 2011), making it especially important to assess when in development children acquire a receptive understanding of the count procedure and how we might aim to improve this understanding. Some research indicates that exposure to “number talk” in the homes of toddlers and older children may play a critical role in the acquisition of these counting abilities (Gunderson and Levine, 2011), but whether numerical input in infancy is similarly important for numerical development is unknown. Given that nothing is known regarding how early (in development), and in what manner, parents begin to talk about number with their young children, it is unclear how to begin assessing the impact of number talk in younger populations. In the current study, we aimed to provide a baseline measure of the level and nature of numerical input that parents may provide to their 5–10 months old infants in the context of reading books. In doing so, this study provides the first exploration of when and how parents spontaneously speak with their young infant about numerical content while also setting the stage for future studies exploring the impact of numerical training in this age group.

### Background

Substantial evidence suggests that numerical input is critical to the advancement of early counting understanding. Correlational evidence indicates that the amount of number talk in the home during toddlerhood predicts cardinal knowledge in the preschool years (Levine et al., 2010; Gunderson and Levine, 2011), suggesting that early numerical input may play a key role in facilitating the count acquisition process. Similarly, the frequency with which slightly older children (grade K and up) engage in both formal (i.e., direct instruction) and informal (e.g., reading books) learning activities in the home predicts later academic numeracy outcomes (Melhuish et al., 2008; LeFevre et al., 2009, 2010; Lukie et al., 2014). Importantly, experimental studies have confirmed that adult numerical input is key in promoting children’s understanding of cardinality. For example, Mix et al. (2012) found that 3.5-year-old children demonstrated marked improvements in cardinal knowledge following 6-weeks of training during which the experimenter labeled and then counted the cardinality of sets in a counting book. Similarly, Posid and Cordes (under review) found that modeling appropriate labeling and counting behavior for only a few minutes increased the likelihood that preschoolers overtly counted while engaging in numerical tasks, resulting in dramatic improvements in performance in two distinct cardinality tasks (relative to children who did not experience the modeling).

Exposing young children to counting is therefore important for furthering their understanding of cardinality and the count procedure. However, despite the importance of numerical input, whether parents actively engage in number talk and what the content of this number talk is early in development remain open questions. Reading is one such method to expose young children to numerical knowledge. In fact, reading is believed to promote the association between spatial and numerical representations among infants (McCrink and Opfer, 2014). This is significant as research indicates that children who begin formal education in kindergarten with a greater knowledge of non-symbolic – or pictorial – representations of number are more likely to demonstrate a better understanding of symbolic mathematics, including number words, at the end of the kindergarten year (Gilmore et al., 2010). Further research indicates that not only do parents read with infants (Murphy, 1978; Karrass et al., 2003) but also engaging in this activity has long-term positive effects for multiple outcomes including academic achievement and language development (Payne et al., 1994; Bus et al., 1995; Sénéchal et al., 1995; Jordan et al., 2000).

In order to determine the importance of reading in introducing young children to mathematical knowledge, Mix et al. (2012) examined whether or not parents spontaneously modeled counting behavior when reading counting books to their preschoolers. They found that when reading a counting book, the majority (69%) of spontaneous utterances made by parents were numerical in nature (e.g., encouraging the child to count, labeling the cardinality of a set, counting, etc.). However, most of these utterances involved asking the child to perform a numerical-based behavior, such as asking the child how many items were present and/or encouraging the child to count. Conversely, very few of the utterances involved the parent demonstrating these numerical behaviors for the child (i.e., counting to the child). Given that preschoolers are in the process of learning these numerical skills, parents were likely more interested in having their child practice these new skills than in providing numerical input that they expected their child to have already mastered.

If parents rarely model labeling and counting behaviors for their preschoolers because they perceive their children as numerically competent, then are they more likely to do so for younger children who are perceived to be less competent? Slaughter et al. (2011) found that 18 months old infants distinguished between an appropriate counting sequence and one in which the one-to-one principle of counting was violated (i.e., some items in a set were counted multiple times whereas other items were never given a counting tag), suggesting that even before children begin to overtly count themselves, they may have a receptive understanding of how the counting procedure works. Presumably, infants must have acquired this knowledge from observing verbal counting, likely via overt demonstrations by their parents early in development. If so, then exposure to number talk and verbal counting behaviors may be more pronounced in infancy than later in development, setting infants on the path toward acquiring numerical concepts.

On the other hand, parents may be less inclined to talk to their infants about number if they assume that preverbal infants are not at a point in development at which they are capable of grasping such an abstract concept (in line with prominent theories of numerical development (e.g., Piaget, 1952; Mix et al., 2002). In support of this account, Levine et al. (2010) reported an increase in parental number talk to toddlers between the ages of 14 to 30 months of age. If so, it may be that even when placed in a context in which numerical information is salient, parents may choose to focus on other more tangible qualities of the items in front of them (e.g., the color or identity of objects), making the level of exposure to numerical concepts a very rare occurrence early in development.

Additionally, numerical input from parents must also vary as a function of set size, with some set sizes highlighted more than others. Clearly, as sets become very large (e.g., 30 items), it is less probable that parents will enumerate individual items within the set to their infant, but are they just as likely to count e.g., two items as they are to count 5? Longitudinal data reveal that the amount of number talk in the home referring to large sets (4–10 items) is a better predictor of cardinal number knowledge later in childhood than number talk referring to small sets (1–3 items; Gunderson and Levine, 2011) but it is unknown whether this relationship extends into infancy. On the one hand, if parents do not view their infants as numerically competent, they may be more likely to count small sets (<4) than larger ones so as not to overwhelm their infants. On the other hand, evidence that adults are able to rapidly apprehend the numerosity of small sets without counting (i.e., subitizing; Mandler and Shebo, 1982; Trick and Pylyshyn, 1994), may make them less inclined to count small sets and only invoke the laborious counting process when the numerosity of the set is not readily apparent (when sets are ≥4). Recently, Mix et al. (2012) reported that parents were significantly more likely to count the sets 4–10 than they were to count set sizes 1–3 to their preschoolers. Whether this was because parents expected their preschoolers to have already mastered small sets and thus focused on larger sets in order to challenge their children, and/or because parents subitized smaller sets and only felt compelled to individuate items in larger sets cannot be determined. Exploring spontaneous counting behavior in parents of younger, less numerically competent, children should both determine the content of numerical input in infancy while also speaking to factors that may compel parents to count a particular set.

In the current study, we extended previous work conducted by Mix et al. (2012; Experiment 2) to examine how parents interact with their infants – across a developmental range (from 5 to 10 months) – in the context of reading an age-appropriate counting book and a similar book that was not numerical in nature. Similar to Mix et al. (2012), we brought parent-child pairs into a quiet room and asked them to read both a counting book (*Cleo’s Counting Book;* Blackstone, 2007) and a non-counting book (*I Love You So…*; Richmond, 2002) as they would at home. We then evaluated the number of spontaneous numerical and non-numerical statements expressed by parents, as well as the amount of time infants spent attending to each book. Parents also completed questionnaires assessing their attitudes and expectations regarding the effectiveness of reading to their children. By conducting this study we hope to learn whether parents spontaneously engage in a potentially educational activity of providing number talk to young infants and how the likelihood of engaging in this activity may change over the course of development in infancy and as a function of set size.

We were interested in determining the following: (a) whether or not infants (as young as 5 months of age) are interested in the presented numeric and non-numeric reading material, (b) whether or not parents’ reading strategies evolve across development or as a function of set size, (c) whether parental reading strategies or expectations are predictive of infants’ level of interest in the material presented to them, and (d) in what contexts do parents engage their infants in numerical conversations? Does this type of interaction occur only while parent are directly prompted with numerical information, or also while reading books that do not highlight number and counting?

## Materials and Methods

### Participants

Participants included 42 infant/parent dyads (35 mothers), recruited when the infant was between the ages of 5–10 months. Participants were recruited via mailings and phone calls to parents of infants born within a 20-mile radius of our main campus, with contact information obtained via public birth records. The racial ethnic break-down was as follows: Caucasian (*n* = 36), Asian (*n* = 2), Black/African American (*n* = 1), Race not indicated (*n* = 3). Additionally, 4 identified as Hispanic, 35 as Non-Hispanic, and 3 did not disclose this information. Although all parents reported that English was the primary language spoken in the home, 24 children came from multi-lingual households. The majority of parents reported having attained a graduate degree (*n* = 30) with the remaining having their highest level of education be a college degree (*n* = 9), high school diploma (*n* = 1), or did not report their education level (*n* = 2). All parents provided consent for themselves and their infants in accordance with the Institutional Review Board’s procedures.

The sample was divided into two groups based upon previous research indicating an average age of onset of reading to infants is 7.6 months (DeBaryshe, 1993). These groups were categorized as “Younger Infants” (5–6 month olds: *M* = 189.5 days, *N* = 22; 13 females) and “Older Infants” (7–10 months: *M* = 269.6 days, *N* = 20; 11 females). Because the purpose of the study was to compare reading behavior when reading a counting book to behavior when reading a non-counting book, to be included in data analyses, the parent had to read through both books. An additional 36 dyads were excluded from the study due to: (1) failure to read through at least one book (*N* = 15) or both books (*N* = 18; completed only one book) due to becoming irritable^1^, (2) equipment (video/audio) failure (*N* = 2), or (3) experimenter error (*N* = 1). Infants in both age groups were equally likely to not make it through both books (14/33 or 42% of non-completers were classified as “Younger Infants”).

### Materials

Parents were given two board books simultaneously, one Counting book, emphasizing counting from 1 to 10 (*Cleo’s Counting Book* by Blackstone, 2007) and one Non-counting book, without a numerical emphasis (*I Love You So…* by Richmond, 2002). Both books were visually similar, such that each featured equally detailed, bright and colorful images that would be interesting to infants and both were made of hard-pages that were similarly sized. Importantly, the books were also of similar length (Counting Book: 22 pages; Non-counting Book: 24 pages). The counting book was selected as it devoted one individual page to each set size between 1 and 10 and the items within the sets were clearly delineated objects. There was no particular narrative to the counting book. On the first page, the text read “Let’s count with Cleo from 1 to 10. Then let’s count back to one again.” Then, on each page, Cleo (a cat) was depicted near a set of objects, and the text generally consisted of labeling the cardinality of a set of items going from 1 to 10 (e.g., “Six tall trees” and “Eight buzzing bees”)^2^. After the page depicting a set of 10, there were two final pages that involved counting up from 1 to 10 and then counting backward from 10 to 1. The text of the Non-counting book was a rhyming poem (e.g., “I love you as loud as a thundery sky, and as tall as the mountains, I love you that high.”). These books were selected because they contained equally colorful and engaging illustrations and yet are less widely known, and as such, they were less likely to be familiar to parents and infants in the study. Of the 38 parents who responded to our questionnaire, only one parent reported being familiar with the counting book and five parents reported being familiar with the non-counting book.

### Procedure

Parents were recruited to visit the lab with their infant for a single-visit study. Parents first completed consent forms and filled out a brief questionnaire regarding their reading habits at home with their infant (e.g., How often do you read to your infant? Is reading part of your daily routine?) while their infant became accustomed to the lab setting. Parents and infants were then brought into the testing room (a well-lit soundproof room), where infants were seated on their parent’s lap. Parents were handed both books (Counting: *Cleo’s Counting Book*, Blackstone, 2007; and Non-counting: *I Love You So…*, Richmond, 2002) and were instructed to read the books to their infant naturally, “just as they would at home.” Parents were told to read through both books in either order and were not instructed which one to read first. The study ended when the parents finished reading both books, or if the parent indicated that they were done (i.e., if the infant was irritable). This procedure was videotaped via a camera mounted in one of the walls of the room, directly in front of the parent-child dyad. Videos were then coded oﬄine.

Although all recruitment materials (flyers, calling scripts, etc.) highlighted the focus of the lab as being on numerical cognition, parents were not specifically told that the books would contain numerical content, nor were they informed that the focus of the study was on exploring parent’s behaviors when reading a counting book.

### Data Coding

Videos were coded oﬄine in 100 ms frames using the Preferential Looking Coder (Libertus, 2008). Videos were coded for the amount of time the parents spent reading each book and the time the infant spent looking at the books. A second independent coder coded infant looking times and parent reading times in a little over 15% of the videos and inter-coder reliability [(# of agreements)/(# of agreements + # of disagreements)] was found to be 94.4% (range 90.5–98.6%).

Additionally, transcripts of the videos were coded for *spontaneous* parental utterances, defined as any meaningful statement made by the parent to the infant that was not specifically printed within the book (i.e., filler utterances such as “uh” or “yeah” that were not meaningful within the context were not counted). These utterances were coded as “numerical” or “non-numerical.” Numerical utterances were further classified as either: (A) Counting (counting out the items on a particular page, but not labeling the cardinality of the set e.g., “One, two, and three”); (B) Labeling (labeling the cardinal value of the array on a page, without counting: e.g., “Look, it’s three!”); (C) Labeling and Counting (labeling the cardinality and counting the items in the set, in either order); or (D) Other (numerical statements that did not fall within either of the two previous categories, e.g., “What comes after four?” or “This is a book about numbers!”). An entire count sequence was classified as a single individual utterance such that a sequence such as “One, two, and three” was coded as one numerical utterance rather than as three discrete ones. Any statement not involving numerical language (utterances not involving number words (e.g., “three”), or the words “count” or “number”) was coded as a non-numerical utterance (e.g., “What’s the kitty cat say?”). On two occasions, a parent read the text of the book and then repeated it again while talking to their infant. These repetitions were treated as spontaneous utterances and coded for numerical content.

A second independent coder coded all transcripts. Inter-rater reliability was again computed as exact agreement = [(# of agreements)/(# of agreements + # of disagreements)] for each different type of utterance during the reading of each book. Reliability was found to be as follows: (1) Counting Book (*M* = 92.3%): (a) Non-numerical utterances - 85%, (b) Numerical utterances - 93%, (c) Counting - 96%, (d) Labeling - 93%, (e) Other Numerical - 89%, (f) Label and Count - 98%; (2) Non-counting Book (*M* = 93.2%): (a) Non-numerical utterances - 85%, (b) Numerical Utterances - 89% (c) Counting - 100%, (d) Labeling - 98%, (e) Other Numerical - 87%, (f) Label and Count - 100%.

Moreover, to verify that our transcriptions were accurate, just over 20% of the videos were transcribed a second time and coded for the utterances described above by a third independent coder. Inter-coder reliability between this third coder (using the second transcripts) and the primary coder (using the original transcripts) across all utterance types and across both books was found to be 94.3% (range 80–100%). Because utterance data were non-normally distributed, these data were log transformed for purposes of statistical analyses.

## Results^3^

### Overall Reading Behavior

Despite being handed both books simultaneously (with side of presentation of each book randomized across parents), parents were significantly more likely (69%) to read the Non-counting book first than the Counting book [29/42, exact binomial *p* (2-tailed) = 0.01]. First book choice, however, did not differ consistently as a function of infant age or sex (*p*’s > 0.1), and none of the dependent variables (e.g., total duration of time spent reading the book, number of spontaneous utterances, or infant looking behavior) differed as a function of book order (*p*’s > 0.05).

A mixed measures ANOVA was conducted to explore whether infant age (Younger, Older) or Book Type (Counting, Non-counting) contributed to differences in the total duration of time parents spent exploring the books with their infants. Analyses revealed that parents dedicated more time to reading and discussing the Non-counting than to the Counting book [*M*_Non-Counting_ = 194.2 s vs. *M*_Counting_ = 163.7 s; *F*(1,40) = 7.26, *p* = 0.01, $\eta_{p}^{2}$ = 0.154], likely due to large differences in the amount of written text in the two books (Non-counting = 309 words; Counting = 147 words). No other significant main effects or interactions were found (*p*’s > 0.13).

Despite differences in the total time spent reading each book, infant looking behavior did not reveal any overt preferences toward one book or the other, appearing to find both books equally interesting. A similar mixed-measures ANOVA looking at the within-subject factor of Book Type (Counting or Non-counting) and the between-subjects factor of Age Group on the proportion of time that infants spent looking at the books (i.e., total duration of looking at books/total time parent read the book) revealed that infants spent a comparable proportion of total time reading with parents looking at the Counting book as they spent looking at the Non-counting book [0.797 vs. 0.787, respectively, *F*(1,40) = 0.112, *p* > 0.7]. Of particular note, no other significant main effects or interactions were obtained (*p*’s > 0.2), revealing that infants across the age range showed relatively comparable rates of attention to the books.^4^ Importantly, although parents spent as long as an average of 3 min reading each book (*M* = 178.7 s per book), infants included in the study found both books interesting and were able to maintain their attention for the duration of the study, spending the majority of the time (79.2% of the time) looking at and engaged in the books^5^.

### Parent Talk: Analysis of Spontaneous Utterances

A mixed measures ANOVA examining the within-subject factors of Book (Counting, Non-counting) and Utterance Type (Numerical, Non-Numerical) and the between-subjects factor of age group on the number of spontaneous utterances made by parents revealed a main effect of Utterance Type [*F*(1,40) = 185.74, *p* < 0.001, $\eta_{p}^{2}$ = 0.823] revealing that, across both books, parents made significantly more non-numerical utterances (*M* = 15.1) than numerical utterances (*M* = 2.6). Moreover, despite spending significantly more time reading the Non-counting book, a main effect of Book [*F*(1,40) = 30.28, *p* = 0.000, $\eta_{p}^{2}$ = 0.431] revealed parents made more spontaneous utterances when reading the Counting book (*M* = 10.1) than the Non-counting Book (*M* = 7.6). However, a Book × Utterance Type interaction [*F*(1,40) = 34.76, *p* = 0.000, $\eta_{p}^{2}$ = 0.465] revealed that parents made a comparable number of non-numerical utterances while reading both books [*M*_Counting_ = 15.3; *M*_Non-counting_ = 14.9; *t*(41) = 0.53, *p* = 0.6], but they made significantly more numerical utterances while reading the Counting book [*M*_Counting_ = 4.9; *M*_Non-counting_ = 0.42; *t*(41) = 6.89, *p* = 0.000]. Thus, despite the relative dearth of numerical talk altogether, parents were significantly more likely to use numerical language when reading the Counting book than when reading the Non-counting book. This finding was also confirmed via non-parametric statistics: whereas only a minority of parents made any numerical statements when reading the non-counting book, most parents did do so when reading the counting book [Non-counting Book: 10/42; Counting Book: 33/42; χ^2^(1, *N* = 84) = 25.21, *p* < 0.001].

**FIGURE 1:**
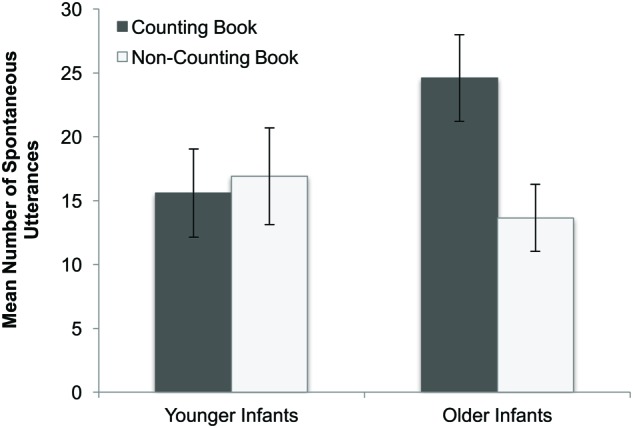
**Mean number of parental spontaneous utterances made as a function of infant age and book type**. Error bars indicate standard errors.

Lastly, the ANOVA revealed a Book x Age Group interaction [*F*(1,40) = 11.39, *p* = 0.002, $\eta_{p}^{2}$ = 0.222] indicating that while parents of younger infants made a comparable number of total spontaneous utterances (numerical and non-numerical combined) while reading the Counting and Non-counting books [*M*_Counting_ = 15.6; *M*_Non-counting_ = 16.9; *t*(21) = 0.02, *p* > 0.9], older infants heard significantly more spontaneous utterances when being read the Counting book [as compared to the Non-counting book; *M*_Counting_ = 24.7; *M*_Non-counting_ = 13.7; *t*(19) = 3.26, *p* = 0.004; see **Figure 1**]. No other significant main effects or interactions were obtained. Together, results reveal that the Counting book prompted parents to use more numerical language and also stimulated parents of older infants to talk more in general.

### Numerical Utterances during Counting Book^6^

Despite visiting a lab that overtly studies infant numerical abilities and being given a book specifically focused on counting, parents rarely spontaneously talked about number to their infants during our study. Of the 837 total spontaneous utterances recorded across all 42 dyads while reading the counting book, only 200 (23.9%) were numerical in nature – dramatically less than the reported proportion of numerical utterances made when parents read counting books to their preschoolers [69% of 220 spontaneous utterances (across 14 dyads), Mix et al., 2012; χ^2^ (1, *N* = 1057) = 160.21, *p* < 0.001]. Thus, in contrast to findings with older children, the majority of spontaneous utterances made by parents while reading the Counting book were non-numerical in nature (average per parent: *M*_Numerical_ = 4.8; *M*_Non-numerical_ = 15.2; *t*(41) = 7.01, *p* = 0.000], with parents preferring to elaborate about the specific items depicted in the book (e.g., “Look at the chicks!”) as opposed to highlighting the number of items present (presumably the purpose of a counting book).

Despite the rarity of spontaneous numerical utterances offered by the parents when reading the counting book, systematic patterns were observed. Consistent with the finding that parents made fewer *total* spontaneous utterances when reading the Counting book to younger infants, analyses revealed that parents also made significantly fewer spontaneous *numerical* utterances when reading to younger, as opposed to older, infants [*M*_Y ounger_ = 2.41; *M*_Older_ = 7.25; *t*(41) = 2.90, *p* = 0.006]. However, developmental differences were not found for the number of non-numerical utterances made during the same period (*p* > 0.11). Importantly, this difference in the number of numerical utterances could not be accounted for by the difference in the total duration of time that parents spent reading the Counting book to younger and older infants, as a similar difference between younger and older infants was found in the *rate* of spontaneous numerical utterances [=number of numerical utterances/total time the parent read the book; *M*_Y ounger_ = 0.017 utterances/sec, *M*_Older_ = 0.038 utterances/sec; *t*(40) = 2.90, *p* = 0.006]. A comparable analysis looking at the rate of non-numerical utterances did not reveal age differences, however, (*M*_Y ounger_ = 0.084 utterances/sec, *M*_Older_ = 0.095 utterances/sec; *t*(40) = 0.64, *p* > 0.5]. Thus, parents did not prioritize numerical input when reading to younger infants, but rather they devoted less time to the Counting book and made relatively fewer numerical utterances than parents of older infants.

**FIGURE 2:**
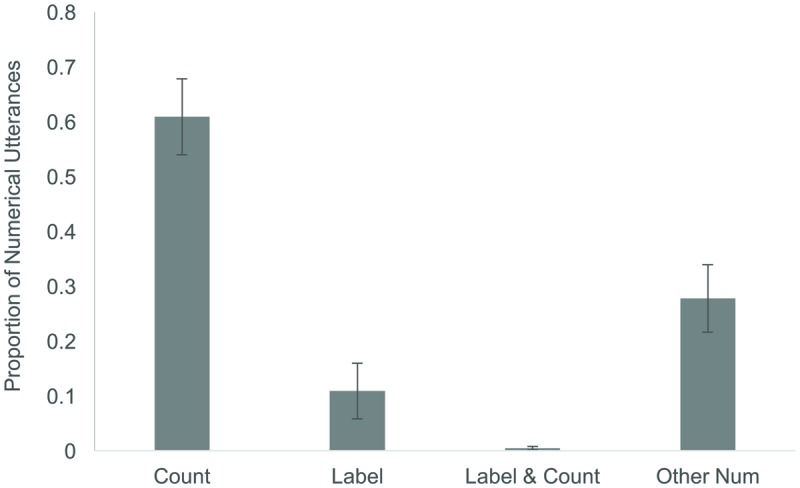
**Proportion of spontaneous numerical utterances made by parents while reading the counting book that was classified as Counting (but not labeling), Labeling (but not counting), Labeling and Counting together, or as Other Numerical Utterances**.

**FIGURE 3:**
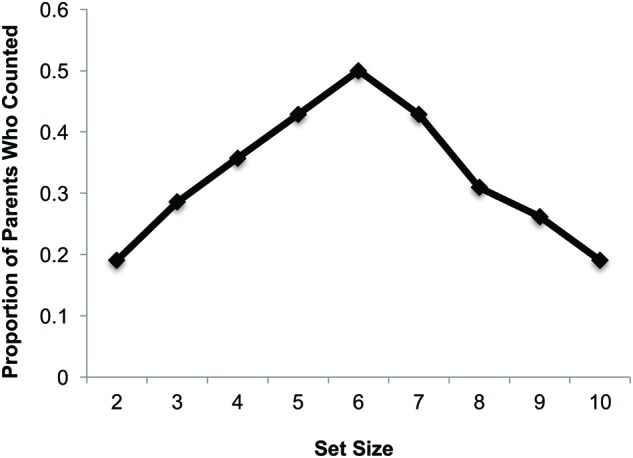
**Proportion of parents who explicitly counted items in the set as a function of the set size**. (Note: the set size of one was not included in this analysis.)

Not surprisingly, the majority of numerical utterances involved counting items in the book (125/200 = 62.5%), with fewer instances of purely labeling the cardinality of the set (9/200 = 4.5%; see **Figure 2**). Parents generally did not spontaneously label *and* count sets at the same time, with only four instances of this type of utterance observed across all parents. Although counting was the most frequent type of numerical utterance, since numerical utterances were so rare, this meant on average, parents counted only 2.95 (*SE* = 0.49) of the nine sets depicted^7^. An Age × Set Size (9) ANOVA revealed a main effect of age [*F*(1,40) = 6.03, *p* < 0.02, $\eta_{p}^{2}$ = 0.13] such that parents of older infants counted more sets than parents of younger infants, and a main effect of set size [*F*(4.8,191.5) = 5.10, *p* < 0.00, $\eta_{p}^{2}$ = 0.113]. Follow-up analyses revealed an inverse U-shaped function relating the probability of a parent counting as a function of set size, with parents most likely to count the set size of 6 and least likely to count the smallest and largest sizes (2 or 10; see **Figure 3**).

### Survey Measures

Of the 42 parent-infant dyads that successfully read through both books, 38 parents completed the survey. Consistent with our expectations, parents in this population generally reported that they read frequently to their infant at home, with 89.5% (34/38) of parents reporting that reading to their child was part of their regular daily routine and 94.7% (36/38) of parents reporting reading 3 or more times per week to their child (*M* = ∼7.25 times per week, estimated based on categories provided by our survey). The frequency of reported reading in the home differed as a function of the infant age group [*F*(1,34) = 6.79, *p* = 0.014, $\eta_{p}^{2}$ = 0.166] and sex [*F*(1,34) = 4.91, *p* = 0.034, $\eta_{p}^{2}$ = 0.126]. In general, parents reported reading more frequently to older infants (*M*_Y ounger_ = 6.06 times/week; *M*_Older_ = 8.16 times/week], consistent with prior research (DeBaryshe, 1993). Parental report also indicated that parents read more frequently to female infants compared to male infants (*M*_Males_ = 6.2 times/week; *M*_Female_ = 8.0 times/week). However, neither parent report of frequency of reading nor parent report of whether they thought their infant enjoyed reading books correlated with infant looking behavior during the study (*p*’s > 0.2) or with any measure of parental spontaneous utterances (*p*’s > 0.18).

## Discussion

The current study is the first to explore parent/infant interactions while reading counting and non-counting books. Results reveal that even as early as the 1st year of life, parents will provide numerical input to their young infants, at least when prompted to do so, when reading counting books. Although they did engage in number talk with their infants, parents did not do so very often, with fewer than one quarter of spontaneous utterances being numerical in nature. While it may seem surprising that parents did not talk about number with their infants more given the context (reading a counting book while being videotaped in a laboratory whose focus is on numerical development), it should be noted that infants in the current study were entirely preverbal, and thus parents may have over-emphasized non-numerical aspects of the books because they may have thought that these concrete concepts (i.e., object labels) were more readily accessible to infants (i.e., their infant is more likely to learn the word “cat” before learning the counting sequence). Regardless, it is remarkable that parents of infants under the age of one still did attempt to talk about number with their infants, suggesting that number talk may begin as early as the 1st year of life.

Moreover, developmental differences were evident. A comparison of the proportion of utterances that were numerical in nature in the current study with that of a different study involving reading to preschoolers (Mix et al., 2012) revealed that infants receive significantly less numerical input than preschoolers when reading counting books^8^. Even more notable was that developmental differences were observed even within the current sample. Whereas infants across the age range heard a comparable number of spontaneous utterances when reading the non-counting book, parents of older infants elaborated much more than parents of younger infants when reading the counting book. As parents on average begin reading with infants at 7.6 months (DeBaryshe, 1993), parents of younger infants may have little experience reading to their infant. As a result, these parents may have reduced utterances due to unfamiliarity with the task. Yet, it is important to note that the difference in utterances could be primarily attributed to a difference in the number of *numerical* utterances provided by parents when reading the counting book; parents of younger infants made significantly fewer numerical utterances than parents of older infants when reading the counting book, but no such differences were found in the number of non-numerical utterances made. Moreover, this difference in numerical input was observed despite the fact that younger and older infants displayed comparable attention and interest in the books, suggesting that parents did not gage the input based upon infant behavior. Thus, it is not that parents simply talk less to their younger infants; instead, it appears that parents specifically talk less about *number* early in development, with the amount of numerical input provided to children by their parents increasing even across the 1st year of life.

Why might this be? One possibility is that this change in the frequency of number talk as a function of infant age reflects a parental sensitivity to the developmental level of their young infant. That is, early in development, parents are likely to emphasize very concrete concepts, such as object labels, for their young infant. However, as infants age, their parents may recognize that their infants may be able to process higher level information and thus begin to incorporate discussions related to abstract concepts, such as set size. Remarkably, our data suggest that a change in parental attitudes toward infant cognition may have been captured even within the small developmental time-window (5–10 months) explored within the current study.

Not only did numerical input vary as a function of the age of the infant, but it also varied as a function of the set size depicted on the page. Parents were most likely to count intermediate set sizes, and less likely to count the smallest and largest set sizes. These findings align with those of a similar study with parents reading to their preschoolers (Mix et al., 2012), who found that parents provided less numerical input for small sets (1–3) compared to large sets (4–10). Mix et al. (2012) attributed this difference to parents expecting their preschoolers to already know the labels for small sets. The fact that parents in our study were less inclined to count the largest set sizes (i.e., 8–10 items) to their preverbal infants may align with a parental-expectation account. That is, parents may not expect their preverbal infants to benefit from watching them count very large sets, and thus chose not to do so. However, if parents were targeting their numerical input toward the abilities of their audience, we would also expect parents in our study to be more likely to count the smallest set sizes (i.e., 2–4 items), since preverbal infants have yet to master these smallest sets and are most likely to learn these set sizes first. Instead, our data, with those of Mix et al. (2012) suggest that parents are less likely to count small set sizes (<4); likely because counting may be less salient to adults for smaller sets because the number of items in small sets may be easily apprehended via subitizing. That is, a comparison of findings across the current study and that of Mix et al. (2012) indicates that the frequency with which parents count a set of a given size does not appear to depend upon the developmental level of the child (since a similar pattern was observed when parents read to infants as when they read to preschoolers), but instead upon the parent’s perceptions of sets of that size.

Importantly, however, the finding that parents were most likely to count intermediate set sizes, which happen to fall within the large number range (4–10 items) may be potentially good news given other evidence suggesting that number talk referring to large sets in 14–30 month olds is a better predictor of later cardinal-number knowledge than number talk referring to small sets (Gunderson and Levine, 2011). That is, if this relationship extends into infancy, then our data suggest that the small amount of number talk that preverbal infants hear in infancy may be focused on larger sets, and thus likely helping to promote the acquisition of later cardinal knowledge. Future work should explore whether number talk around large sets holds a similar place of importance in the numerical development of preverbal infants. For example, highlighting attention to and individuating items within large sets may promote an early spontaneous attention to number in the world around them (i.e., SFON or Spontaneous Focusing on Numerosity; Hannula and Lehtinen, 2005), a trait which has been linked to early counting abilities.

Parents in the current study were asked to read counting books to their infants as it was postulated that this was a context that would promote “number talk” on the part of the parents. Data analyses confirmed this to be the case, at least relative to reading a non-counting book. That is, parents made significantly more numerical utterances when reading the counting book than when reading the non-counting book, and a greater proportion of parents volunteered any numerical information when reading the counting book as compared to the non-counting book. Thus, at least within the context of reading books, counting books did promote the use of number talk, just not enough to make it the primary focus of the conversation.

Parents also made significantly more spontaneous utterances, in general, when reading the counting book compared to the non-counting book. This is not surprising, given that the text of the counting book contained fewer than half as many printed words as the text of the non-counting book. What is remarkable, however, is that parents made a comparable number of spontaneous non-numerical utterances while reading both books – it was only the number of spontaneous numerical utterances that differed across books. Thus, while the relative scarcity of printed text in the counting book may have prompted parents to elaborate more when reading that book, it appears that the primary content of these additional spontaneous utterances made was numerical in nature.

Despite the promotion of number talk in the counting book context, parents still rarely talked about number. Why might this be the case? One possibility is that since the written text of the counting book primarily involved labeling the cardinality of a set (e.g., “Eight buzzing bees”), parents felt as though the numerical aspects of the display had already been addressed and instead preferred to elaborate on the non-numerical aspects. In line with this idea, it may be that parents are more likely to engage in number talk in less-structured contexts, such as playing with toys with their infants. However, if this were the case, parents should have volunteered more numerical utterances when reading the non-counting book, which did not happen. Alternatively, it may simply be that infants in this age range rarely receive numerical input because parents do not expect preverbal infants to benefit from this type of input, thinking they are too young to understand abstract numerical concepts (consistent with Piaget, 1952), preferring instead to focus on more concrete knowledge (e.g., object labels). Future work should investigate whether greater numerical talk is found during less-structured parent/infant interactions, as well as look into parent attitudes toward infant numerical competencies and their relation to the frequency of numerical input, to explore this question further.

It is important to note that, given the young age of the infants involved and the lengthy task of reading two books, our study had a fairly high attrition rate relative to that of other studies. The analyses in this study were conducted only on data collected from parent/infant dyads who were capable of successfully completing the study. It is unknown whether this sample of participants is generalizable to the general population or if, for example, the infants in this sample may have had higher than average attention spans and/or a calmer temperament than others. Additional investigations into this area, perhaps requiring the reading of only a single counting book, are thus warranted.

Clarifying the content and amount of number talk provided to infants is an important step toward understanding the development of numerical abilities. Studies reveal that infants are sensitive to numerical information, regularly discriminate between sets based upon number, and even have a coarse understanding of the counting procedure (e.g., Xu and Spelke, 2000; Wood and Spelke, 2005; Cordes and Brannon, 2008, 2009; Libertus and Brannon, 2010; Slaughter et al., 2011). Moreover, numerical abilities as early as 6 months of age have been shown to predict mathematics achievement in the preschool years (Starr et al., 2013). Thus, even within the 1st year of life, infants are tuned into numerical information and likely to be receptive to numerical input in their environment. Given that number talk (including counting) has been shown to promote numeracy in young children (Melhuish et al., 2008; LeFevre et al., 2009, 2010; Levine et al., 2010; Mix et al., 2012; Lukie et al., 2014; Posid and Cordes, under review), it is possible that preverbal infants may similarly benefit from numerical input. While currently there is not enough research to conclude whether or not number talk in the 1st year of life may impact a child’s numerical abilities, the current study sets the stage for future research to explore the importance of this early number talk. The current study established a baseline measure of the amount of number talk parents engage in with their young infants. Thus, future studies can explore the role of this type of early input in the development of early numerical abilities and determine whether highlighting numerical information to infants may promote later math achievement.

## Conclusion

Results reveal that even in the context of reading counting books, preverbal infants receive strikingly little numerical input from their parents. The numerical input they receive, however, appears geared toward the situation (reading a counting book vs. reading a non-counting book) and toward the age of the infant (with younger infants receiving less numerical input than older infants). These developmental differences likely demonstrate a shift in parental attitudes toward infant numerical competencies within the second half of the 1st year of life. Together, results serve as an initial step toward clarifying the role parents may play in helping preverbal infants learn about number in the world around them.

## Author Contributions

AG, TC, and SC contributed through data collection, data processing, coding data, data analysis, and writing of the publication.

## Conflict of Interest Statement

The authors declare that the research was conducted in the absence of any commercial or financial relationships that could be construed as a potential conflict of interest.

## Footnotes

1 Although the length of both books was comparable to that of other popular baby books, it is suspected that infants became irritable at a somewhat higher rate than in other infant studies because infants in our study were generally younger than the toddlers included in other comparable studies (e.g., de Mendoza, 1995) and because parents were asked to read *two* books to their infants (as opposed to one book; e.g., DeLoache and DeMendoza, 1987), which made the average total duration of reading time just under 6 min.

2 On two of the last pages, there was slightly more text involved. On the page depicting a set of 9 items, the text read “Nine fluffy chicks – but where is mother hen?” and on the page depicting a set of 10 items the text read “Here she comes with one more chick – and that makes 10!”

3 All behavioral analyses were initially performed with infant sex as a variable, but no main effects or interactions involving that variable were significant and thus this variable was not included in the main analyses reported here. Survey measures did reveal sex differences and those results are reported here.

4 Attention refers to visual attention alone and not auditory attention.

5 It is important to note that these analyses only include infants who completed the study. A large proportion of the infants became irritable when reading the books and were excluded prior to these analyses.

6 All utterance analyses were re-performed including data from the additional 15 dyads who made it through the counting book but failed to complete the non-counting book and the pattern of results was identical.

7 This excludes the first set containing only a single item, as it is less clear how to determine whether a parent counted one object, and excludes pages at the beginning and end of the book in which the numbers from 1 to 10 were written on the page (since the words were considered part of the text).

8 It is important to note that, in addition to a difference in the age of the children in the studies, there are procedural differences between the Mix et al. (2012) study and the current study that may also account for the lower number of spontaneous numerical utterances obtained in the current study.
